# Telomeres and Telomerase in Cutaneous Squamous Cell Carcinoma

**DOI:** 10.3390/ijms20061333

**Published:** 2019-03-16

**Authors:** Alessandra Ventura, Cristina Pellegrini, Ludovica Cardelli, Tea Rocco, Valeria Ciciarelli, Ketty Peris, Maria Concetta Fargnoli

**Affiliations:** 1 Department of Dermatology, Department of Applied Clinical Sciences and Biotechnologies, University of L’Aquila, 67100 L’Aquila, Italy; alessandraventura@live.it (A.V.); ludovica.cardelli@graduate.univaq.it (L.C.); tearocco@gmail.com (T.R.); valeria.ciciarelli@hotmail.it (V.C.); mariaconcetta.fargnoli@univaq.it (M.C.F.); 2 Institute of Dermatology, Fondazione Policlinico Universitario A. Gemelli-IRCCS, Università Cattolica del Sacro Cuore, 00186 Rome, Italy; Ketty.Peris@unicatt.it

**Keywords:** cutaneous squamous cell carcinoma, telomere, telomerase, *TERT* promoter

## Abstract

The role of telomere biology and telomerase activation in skin cancers has been investigated in melanoma and basal cell carcinoma but limited evidence is available for cutaneous squamous cell carcinoma (cSCC). We will review the current knowledge on the role of telomere and telomerase pathway in cSCC pathogenesis. At the somatic level, both long and short telomere lengths have been described in cSCC. This telomere dichotomy is probably related to two different mechanisms of tumour initiation which determines two tumour subtypes. Telomere shortening is observed during the invasive progression from in situ forms of cSCC, such as Bowen’s disease (BD) and actinic keratosis (AK), to invasive cSCC. At the germline level, controversial results have been reported on the association between constitutive telomere length and risk of cSCC. Approximately 75–85% of cSCC tumours are characterized by a high level of telomerase activity. Telomerase activation has been also reported in AKs and BD and in sun-damaged skin, thus supporting the hypothesis that UV modulates telomerase activity in the skin. Activating *TERT* promoter mutations have been identified in 32–70% of cSCCs, with the majority showing the UV-signature. No significant correlation was observed between *TERT* promoter mutations and cSCC clinico-pathological features. However, *TERT* promoter mutations have been recently suggested to be independent predictors of an adverse outcome. The attention on telomere biology and telomerase activity in cSCC is increasing for the potential implications in the development of effective tools for prognostic assessment and of therapeutic strategies in patients with cutaneous cSCC.

## 1. Introduction

Cutaneous squamous cell carcinoma (cSCC) accounts for 20% of all non-melanoma skin cancers (NMSCs) and is the second most common NMSC in white populations [1,2]. The lifetime risk of developing cSCC is 7–11% in USA [3]. The incidence of cSCC has been increasing in the last decades [4] with rates of 15.8 cases per 100,000 in Europe [5]. cSCC is twice more common in men than in women and the mean age of cSCC occurrence is 70 years. Old age, fair skin type and chronic exposure to ultraviolet (UV) radiation are the major risk factors for the development of cSCC with 90% of the tumours arising on sun-exposed areas such as the head and neck region, dorsum of the hands and the forearms [6,7].

cSCCs can clinically appear as a small plaque or an asymptomatic nodule that increases in size over time, often ulcerating. Other clinical forms of cSCC include Bowen’s disease (BD), which represents an in situ cSCC usually occurring on non-sun exposed areas and the crateriform (keratoacanthoma), ulcerated, necrotic and vegetative subtype [8]. cSCC often arises in the context of actinic keratosis (AK), which can become hyperkeratotic and infiltrating over time [9]. While most cSCCs develop in the context of an AK, the AK progression rate into invasive cSCCs is less than 1/1000 per year over a 5-year follow-up [10]. Prompt diagnosis and surgical removal offer the best chance of cure, with only 5–8% of cSCC patients experiencing metastases [11]. Tumour depth greater than 2 mm, Clark level of IV or more, perineural involvement, primary site on the ear or non-hair-bearing lip and poorly differentiated tumour are considered high-risk features for the development of metastases [12].

As in the majority of other skin cancers, a number of molecular aberrations are required for initiation of the tumorigenic process, including a modified cell cycle control, onset of genomic instability, establishment of a telomere maintenance mechanism, induction of angiogenesis and of a pro-inflammatory environment [13]. The most common somatic mutations in cSCC occur in the tumour suppressor gene *TP53*. *TP53* mutations represent an early event in carcinogenesis since they are frequently detected in AKs and in in situ cSCC. Mutations in the *TP53* gene determine resistance to apoptosis, thus allowing clonal expansion of mutated cells. UVB exposure is responsible for a significant proportion of *TP53* mutations that mainly occur at pyrimidine dimer sites [14]. Moreover, loss of function alterations in tumour suppressor genes, including *CDKN2A* and *NOTCH 1/2* and aberrant activation of *EGFR* and *HRAS* genes, have been reported in cSCC pathogenesis [15]. 

Several studies support the role of telomere biology and telomerase activation in the development of malignant skin cancers. It has been extensively reported in melanoma and, more recently, in basal cell carcinoma (BCC), while limited data are available in cSCC [16,17,18,19,20]. In this review, we will summarize the current knowledge about the role of telomere and telomerase pathway in cSCC pathogenesis.

## 2. Physiologic Role of Telomeres

**Figure 1:**
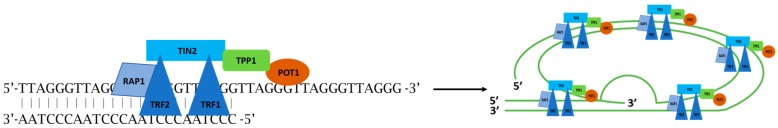
Telomeres are nucleoprotein structures composed of double-stranded short repeated sequences ending in a single-stranded G-rich overhang. Telomeric DNA is coated by specialized proteins that form the so-called shelterin complex. This group of proteins comprises six components: three DNA-binding proteins and three proteins that act as adaptors and mediate interactions among components. TRF1 and TRF2 bind the double strand DNA, while POT1 accumulates at the single-stranded G-rich overhang via TPP1 to protect telomeres. TPP1 connects with TIN2 that plays a role in stabilizing the shelterin complex through simultaneous binding with TRF1, TRF2 and TPP1. RAP1 facilitates the function of TRF2 and improves protection of telomeres through the formation of telomeric loop, T-loop.

Telomeres are nucleoprotein complexes present at the ends of eukaryotic chromosomes with the main role of preserving chromosome integrity and genome stability, avoiding the ends from being recognized by the DNA damage surveillance pathways and undergoing fusion and recombination [21,22]. They consist of several replications of the TTAGGG sequence, accounting for a telomere length of approximately 10–15 kb in humans [23,24]. During the lifetime of a normal somatic cell, DNA polymerase is unable to replicate the final bases prior to cell division, thus determining telomere shortening at each round of cell division and creating a replication limit [25,26,27]. Telomere shortening has been proposed to represent a ‘mitotic clock’ and a cellular mechanism to calculate cell divisions [28] and is thought to protect against malignant transformation. A failure of this process could result in genomic instability and carcinogenesis. Critical shortening of telomeres determines that cells recognize their chromosome ends as double-strand interruptions and the damaged chromosomes might undergo fusion or instability [29,30]. Therefore, telomeres are essentially unstable and represent fragile sites. The shelterin complex binds telomeres and guarantees stability assisting the replication process. It is composed of six subunits, three proteins (TRF1, telomere repeat binding factor 1; TRF2, telomere repeat binding factor 2; POT1, the protection of telomere protein 1) that bind directly to telomeric DNA and three (RAP1, repressor-activator protein 1; TIN2, TRF1 and TRF2 Interacting Nuclear Protein 2; TPP1, TIN2 organizing protein) that mediate the interaction between components [31,32,33]. Shelterin proteins also cooperate with other elements that transiently localize on telomeres in a cell-cycle dependent manner and aid in creating a shielding T-loop at the chromosome ends [32] (Figure 1).

TRF1, telomere repeat binding factor 1; TRF2, telomere repeat binding factor 2; POT1, protection of telomeres protein 1; TPP1, abbreviation derived from TINT1/PTOP/PIP1; TIN2, TRF interacting nuclear factor 2; RAP1, Repressor/activator protein 1.

Constitutive telomere length is a polygenic trait with high estimated heritability and has been associated with nine different genetic loci, six of which harbouring genes related to telomere homeostasis [24]. Two major pathways have been described to maintain telomere length. The first pathway is represented by the activation of telomerase which elongates telomeres by adding hexameric 5′-TTAGGG-3′ tandem repeats to the chromosomal ends and the second one is an alternative, non-telomerase-dependent mechanism known as alternative lengthening of telomeres (ALT) [34,35].

**Figure 2:**
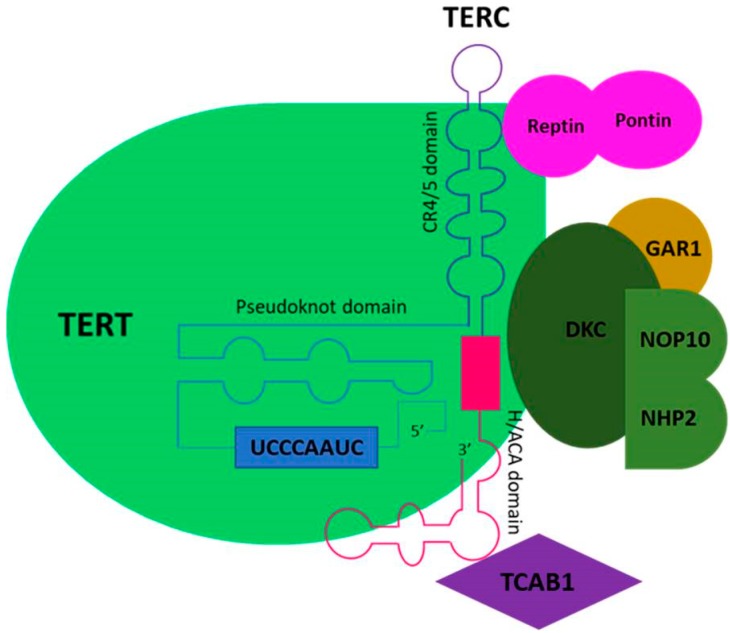
Telomere elongation relies on telomerase, a ribonucleoprotein including a catalytic subunit telomerase reverse transcriptase (TERT) and an RNA component (TERC). Telomerase extends telomeres by adding hexameric 5′-TTAGGG-3′ tandem repeats to the chromosomal ends via reverse transcription using an intrinsic RNA template region (UCCCAAUC). TERT protein comprises anchor sites for telomeric DNA and also contributes to telomere catalysis. TERC component contains three conserved domains: the template/pseudoknot domain, the CR4/5 domain that are required for telomerase activity and the H/ACA domain, conserved in all vertebrates. Maturation of telomerase involves an elaborate number of molecules, among which TCAB1 that recruits mature telomerase complex. Furthermore, in vivo telomerase function needs auxiliary protein components, including DKC, NOP10, GAR1, NHP2, reptin and pontin.

Telomerase is a ribonucleoprotein enzymatic complex involved in the stabilization of telomeres. It is capable of adding telomeric repeats (TTAGGG)n sequences to the ends of chromosomes and therefore of discontinuing shortening of the chromosomes with each cell division [36]. Telomerase is composed by a reverse transcriptase heterodimer formed by a noncoding RNA template (TERC, telomerase RNA component) for de novo synthesis of telomeric DNA sequences and by an enzymatic subunit (TERT, telomerase reverse transcriptase) [37]. Dyskerin, Telomerase Cajal body protein 1 (TCAB1) and nuclear protein family A, member 3 (NOP10) are associated with the telomerase complex and play important functions in telomerase recruitment to telomeres and in the subcellular localization of the telomerase complex [38,39] (Figure 2).

TCAB1, Cajal body protein 1; DKC, dyskerin; NOP10, NOP10 ribonucleoprotein; GAR1, GAR1 ribonucleoprotein homolog; NHP2, NHP2 ribonucleoprotein.

Telomerase activation is predominantly regulated at the transcriptional level. The *TERT* gene is situated on chromosome 5p15.33. The core promoter region of this gene consists of 260 base pairs with several binding sites that regulate gene transcription [20]. In addition, *TERT* expression is regulated by c-Myc, Mad1, the receptors for the hormones estrogen and progesterone, AP-1, NF-kB, Rb/E2F factors, CEBP-alpha and CEBP-beta [40,41]. Moreover, the Wnt/beta-catenin pathway and the KLF4 were reported to regulate *TERT* gene expression and telomerase activity in stem cells [42,43].

## 3. Telomere Biology in Cancerogenesis

The role of telomere biology in tumorigenesis is complex and influenced by multiple mechanisms. Molecular alterations of telomere and telomerase pathways play a pivotal role in cellular immortalization, tumorigenesis and progression of cancer.

The majority of cancerous tissues have short telomeres [44]. Telomere shortening is a natural consequence of cell division that trigger cellular senescence. Moreover, it is influenced also by oxidative damage and replicative stress caused by genetic, epigenetic and environmental factors [24]. In the absence of a signal for repeated elongation, telomere shortening acts as a tumour suppressor mechanism. However, in cells undergoing replicative senescence, the gain of oncogenic changes (i.e., in p53 and p16) could allow to bypass senescence and induce cellular divisions until multiple telomeres critically shortened (telomere crisis) [20]. This induces chromosome breakage-fusion-bridge cycles that bring genomic instability and extensive cell death. In this context, rare cells can bypass the crisis and become immortal by the reactivation of telomerase or by ALT mechanism [22]. Therefore, genomic instability, a basic hallmark of most cancers, would be more evident with short telomeres.

The relation between shortened telomere length and cancer has been investigated in several studies [45]. Shorter telomeres have been reported in bladder, esophageal, gastric, head and neck, ovarian, renal cell carcinoma and have been associated with poor overall survival [46,47]. However, individuals with constitutively long telomeres and/or variant genes associated with long telomeres exhibit increased risk of major cancers as melanoma, breast, lung adenocarcinoma and prostate cancer [48,49,50]. Increased risk of cancers due to long constitutive telomeres, instead of shorter, has been termed as a paradox [44].

Telomerase activity is gradually downregulated during embryogenesis and is usually suppressed in adult somatic cells, mainly due to repression of *TERT* gene expression [41]. However, adult stem cell compartments and highly proliferating cells such as proliferating B- and T-cells and the regenerating hepatocytes maintain low levels of telomerase activity [51,52,53,54]. Reactivation of telomerase is a key event in carcinogenesis and allows proliferative cancer cells to preserve telomere length [55]. Increased telomerase activity has been described in a high percentage of tumors and transcriptional alteration of the *TERT* gene is the major cause of its cancer-specific activation. In addition to its role in maintaining telomere length, telomerase exhibits multiple biological activities independently of its telomere-lengthening function. Aberrant *TERT* expression was shown to promote malignant transformation by influencing many processes such as cell signaling, proliferation, apoptosis and migration. Recently, TERT overexpression has been demonstrated to promote invasion in cancer cells through the acquisition of an invasive mesenchymal phenotype (epithelial-to-mesenchymal transition) and stem cell-like traits [56,57,58].

*TERT* promoter mutations represent a key mechanism for cancer-specific telomerase activation, especially in cancers developing from tissues with low rates of self-renewal [59]. *TERT* promoter mutations have been found to influence the transcriptional regulation of the *TERT* gene and were identified in several cancers including melanoma, NMSCs, bladder cancer and glioma [17,60,61,62,63].

There is much evidence supporting the influence of telomere length and telomerase activity in the pathogenesis of malignant skin cancers. The incidence of malignant skin cancers is increased in the presence of mutations in genes implicated in the maintenance of telomere integrity [32,64] and among patients suffering from telomere-related genetic syndromes [65]. Telomere length was positively associated with nevus count [66], which is the major indicator of melanoma risk [24]. Germline analysis in melanoma patients demonstrated the role of longer constitutive telomeres in melanoma development [67,68]. Longer telomeres in melanoma families were associated to telomerase reactivation mainly due to *TERT* promoter mutations. On the contrary, at the somatic level, recent evidences demonstrated that short telomeres are related with poor melanoma-specific survival [24]. The explanation might be that longer telomeres allow generous time for cell division thus explaining the association with increased risk of developing cancer. Nevertheless, once a patient has developed the cancer, shorter telomeres would likely lead to rapid chromosomal fusions and aneuploidy determining a poor outcome [69]. An increased risk of BCC was associated with shorter constitutive telomeres [70], although at the somatic level BCC telomeres were not found to be shortened in comparison to control epidermis [71]. Recent studies identified a high prevalence of *TERT* promoter mutations in BCC tumors [72]. The majority of *TERT* promoter mutations present an UV-signature with C > T or CC > TT modifications, supporting a pivotal role of UV exposure.

## 4. Telomere and Telomerase Activity in Cutaneous Squamous Cell Carcinoma

cSCCs are characterized by a high frequency of chromosomal aberrations and a high level of genomic instability [73], which might be related to telomere biology alteration [74]. However, it is still debated how telomere biology and telomerase activation contribute to tumor development (Table 1).

### 4.1. Telomere Length

Preliminary evidences that telomere length might be involved in cSCC development come from studies performed in transplanted patients. In this first study, telomeres were shown to be consistently longer in BD and cSCC tumors of transplanted patients as compared to those of non-transplanted patients and were shorter in tumor specimens as compared with their matched normal skin [75].

Leufke et al. (2014) evaluated the telomere profile in cryopreserved cSCC tissues demonstrating that telomere length determines two tumor subtypes, one exhibiting short/medium telomeres with a homogeneous size distribution across the tumor and the other one characterized by long telomeres associated with heterogeneous size variation [76]. Interestingly, this telomere dichotomy was observed in cSCC from renal transplant recipients, suggesting that both telomere subtypes contribute also to cSCC under immunosuppression. Demographic and clinical features such as tumor histotype, location, patient’s sex and age failed to discriminate the two cSCC telomere phenotypes. This telomere dichotomy was not limited to cSCCs but was already observed in AKs, suggesting that AKs are the precursor lesion of both cSCC subtypes and confirming two potential different mechanisms of tumor initiation in cSCC [76]. It has been hypothesized that AKs and SCCs characterized by a homogeneously short/intermediate telomere phenotype originate from stem cells in the basal layer which is considered as the tumor-initiating cell. On the other hand, it remains unclear by which event and from which cell the long/heterogeneous telomere phenotype originates. Finally, cSCC with a heterogeneous telomere phenotype generally exhibited more genetic aberrations, with multiple chromosomal gains as well as frequent loss of genetic material, compared to those with the homogeneous profile. A higher degree of aberrant p53 and cyclin D1 expression has been related to long/heterogeneous telomeres [76].

The relation of telomere length with the malignant potential of different NMSCs was analyzed in AK, BD, BCC and cSCC tumours and surroundings epidermal tissues by calculating the telomere centromere ratio (TCR), which reflects telomere length more accurately than other experimental procedures [77]. TCR values for cSCC were significantly lower than those for BD and AK (cSCC < BD < AK) and peritumoral epidermal cells had higher TCR values than tumour cells. These findings demonstrated that tumour biological behaviour was intrinsically related to telomere length and that telomere shortening is consistent with the invasive progression [77].

Regarding constitutive telomere length at the germline level, the photoaged phenotype of patients with cSCC is expected to reflect an increased senescence and therefore shorter telomeres [66]. Results of three independent studies on constitutive telomere length and risk of cSCC were recently reviewed and a clear association was not found [78]. Han analysed constitutive telomere length by quantitative real time PCR in a nested case–control study, including 254 cSCC cases and 273 controls. They did not observe a significant association between relative telomere length and cSCC risk [70]. Similar findings were reported by Liang and colleagues in 241 cSCC cases and 241 controls [79]. Contrariwise, longer telomeres were inversely associated with cSCC risk in a clinic-based case-control study evaluating 136 cSCC patients and 372 controls [80]. Long telomeres were shown to be protective for cSCC, with a OR of 0.01 [95% CI: 0.00–0.05] [80]. A cumulative summary relative risk was not calculated due to the very large heterogeneity between the three studies [78].

### 4.2. Telomerase Activity

Activation of the telomerase enzyme is considered an essential step in skin carcinogenesis. 

A high telomerase activity was reported in 75–85% cSCCs, with no association with tumour histotype, location or aggressiveness and was also detected in peritumoral skin, although its level was significantly lower than in affected skin [18,81]. Another study reported opposite results with a high telomerase activity in only 25% of cSCCs. These authors hypothesized that the low enzymatic activity might be related to the limited aggressiveness and low metastatic rate of the majority of cSCC included in their study [19]. Interestingly, no association between telomerase activity and telomere length was found. When cSCC was compared to BCC, a higher level of telomerase activity was observed in cSCCs than in BCC speculating that this might explain the different malignant potential of these tumour types [18,82].

Telomerase activity was found to be elevated in AKs and in sun-damaged skin compared to sun-protected sites, thus supporting that UV modulates telomerase activity in the skin [16]. In line with these findings, telomerase activation was shown to be involved at an early stage during cSCC carcinogenesis, since it was detected not only in cSCCs but also in AKs and BD and preceded the occurrence of UV-associated p53 mutations in the skin [81].

TERT is the rate-limiting component of the telomerase complex. The change in TERT expression during the different steps of skin carcinogenesis was analysed in keratoacanthomas (KA), as a paradigm for early tumour and in cSCCs, as the representative of invasively growing late-stage tumour. The immunochemical analysis of TERT expression showed no significant difference between KA and cSCC tumours. Half of the tumours in both groups showed TERT staining in 30–70% of the nuclei and the remaining cases a weak TERT staining. TERT expression did not specifically increase during progression of cSCC, suggesting that telomerase upregulation might not be needed as an additional step during the carcinogenesis process. Notably, in the majority of AK and cSCC the expression was restricted to focal areas, indicating that only certain populations of tumour cells may have gained the ability to express substantial level of TERT [83].

The potential mechanism of TERT re-activation has been attributed to genetic changes within the promoter of the gene that harbours binding sites for numerous transcription factors. *TERT* core promoter mutations at the somatic level have been well described as main responsible for telomerase activation in melanoma and BCC, while only few studies analysed cSCC. Activating *TERT* promoter mutations have been identified in 31.6 % to 70% of cSCC lesions in four different studies, including a total of 239 cases [72,84,85,86]. The most frequently identified mutations were c.−146 C > T and c.−124 C > T. Overall, all mutations showed a UV-signature (C > T and CC > TT) consistent with an etiologic role for UV exposure in the development of cSCC [72,84,85,86]. The mutation rate was higher in invasive cSCC (34.7%) than in in situ cSCC (19.4%) [85]. No significant correlation between *TERT* promoter mutations and clinical pathological features was observed [72]. Interestingly, the prognostic value of TERT promoter mutations has been reported in cSCC, with *TERT* promoter mutations (OR, 8.11; *p* = 0.002) and age > 75 years (OR, 14.84; *p* = 0.013) identified as independent predictors of recurrences and metastases.

At the germline level, the association between 39 SNPs at telomere-related loci, including *TERT*, *TRF1*, *TRF2*, *TNKS2*, *POT1*, *TERT-CLPTM1L* and the risk of skin cancer was investigated in a nested case-control study including 285 Caucasian cSCC patients. No significant association was observed between the genotyped SNPs and cSCC risk [87].

## 5. Conclusions

It is still a matter of debate how telomere biology and telomerase activation contribute to skin carcinogenesis and controversial results have been reported for cSCC, mainly due to limited available evidences.

Two telomere profiles have been described in cSCCs at the somatic level, one exhibiting short/medium telomeres with a homogeneous size distribution within the tumour and the other with a long/heterogeneous profile. This telomere dichotomy is probably explained by two different mechanisms of tumour initiation and is not limited to cSCCs but already observed in AKs. Telomere shortening has been associated with tumour progression, increasing from in situ forms (AK and BD) to invasive cSCC.

At the germline level, the current available literature does not allow to define if there is any association between telomere length and cSCC, with two population-based studies reporting no association and one hospital-based study showing a strong association between decreasing telomere length and risk of cSCC. Telomere length measurement might be a potential risk stratification biomarker for estimating cancer risk.

Telomerase activation was observed in early lesions, AKs and BD, in invasive cSCC and also in peritumoral skin, although its level was significantly lower than in affected skin. In addition, sun-damaged skin showed higher level of telomerase activation compared to sun-protected sites, thus supporting the hypothesis that UV modulates telomerase activity in the skin [16,73].

A potential mechanism of TERT re-activation has been attributed to mutations in the gene promoter. Activating *TERT* promoter mutations have been identified in a high percentage of cSCC lesions, with the majority showing the distinctive UV signature. The mutation rate was higher in invasive cSCC than in in situ cSCC and was recently associated with a poor outcome. Telomerase activity can represent an effective tool for our understanding of cancer etiopathogenesis and in a close future for improving cancer treatment strategies.

The prominent role of telomerase in human cancers has encouraged the development of telomerase inhibitors to suppress tumour growth and gene therapy and immunotherapy have been proposed to potentially control TERT expression in tumours. The development of therapeutic strategies against telomere maintenance cancers might be effective against a vast majority of neoplasias, including cSCC.

## Figures and Tables

**Table 1:** Overview of main findings reported on telomere biology and telomerase activity in cSCC.

Study	Year	Molecular Alterations Investigated	Number of Cases	Main Findings
Taylor, R.S. et al. [18]	1996	Telomerase activity	18	Telomerase activity was detected in 84% of cSCC
Burnworth, B. et al. [83]	2006	TERT expression	31	Telomerase expression is not specifically increased during cSCC malignant progression
Perrem, K. et al. [75]	2007	Telomere length	66	Telomeres were longer in cSCC tumours of transplanted patients as compared to those of non-transplanted patients
Han, J. et al. [70]	2009	Telomere length	285	No association was observed between telomere length and cSCC risk
Liang, G. et al. [79]	2011	Telomere length	241	No significant association was detected between telomere length and risk of cSCC
Griewank, K.G. et al. [72]	2013	*TERT* promoter mutations	34	*TERT* promoter mutations were identified in 50% of cSCC cases
Scott, G.A. et al. [84]	2013	*TERT* promoter mutations	26	50% of cSCC presented classic mutations of *TERT* promoter *
Anic, G.M. et al. [80]	2013	Telomere length	136	Long telomere length was inversely associated with cSCC risk
Leufke, C. et al. [76]	2014	Telomere profile	32	cSCC expressed two telomere phenotypes: (i) tissue-wide short to intermediate telomere length and (ii) tissue-wide heterogeneous longer telomere, suggesting two modes of initiation
Cheng, K.A. et al. [86]	2015	*TERT* promoter mutations	84	TERT promoter mutations were identified in 70% of cutaneous cSCC
Yamada-Hishida, H. et al. [77]	2017	Telomere length	9	Telomere length of cSCC was significantly shorter than those of BD and AK; telomere shortening is correlated with invasive progression
Campos, M.A. et al. [85]	2019	*TERT* promoter mutations	184	TERT promoter mutations are associated with poor prognosis in cSCC

* Classic mutations comprise the most recurrent *TERT* promoter mutations: −146 C > T, −124 C > T and −138–139 CC > TT. AK, actinic keratosis; BD, Bowen’s disease; cSCC, squamous cell carcinoma; TERT, Telomerase reverse transcriptase.

## References

[1] Motley R., Kersey P., Lawrence C., British Association of Dermatologists, British Association of Plastic Surgeons, Royal College of Radiologists, Faculty of Clinical Oncology (2002). Multiprofessional guidelines for the management of the patient with primary cutaneous squamous cell carcinoma. Br. J. Dermatol..

[2] Preston D.S., Stern R.S. (1992). Nonmelanoma cancers of the skin. N. Engl. J. Med..

[3] Miller D.L., Weinstock M.A. (1994). Nonmelanoma skin cancer in the United States: Incidence. J. Am. Acad. Dermatol..

[4] Gloster H.M., Brodland D.G. (1996). The epidemiology of skin cancer. Dermatol. Surg..

[5] Holme S.A., Malinovszky K., Roberts D.L. (2000). Changing trends in non-melanoma skin cancer in South Wales, 1988–1998. Br. J. Dermatol..

[6] Revenga Arranz F., Paricio Rubio J.F., Mar Vazquez Salvado M., del Villar Sordo V. (2004). Descriptive epidemiology of basal cell carcinoma and cutaneous squamous cell carcinoma in Soria (north-eastern Spain) 1998–2000: A hospital-based survey. J. Eur. Acad. Dermatol. Venereol..

[7] De Vries E., Trakatelli M., Kalabalikis D., Ferrandiz L., Ruiz-de-Casas A., Moreno-Ramirez D., Sotiriadis D., Ioannides D., Aquilina S., Apap C. (2012). Known and potential new risk factors for skin cancer in European populations: A multicentre case-control study. Br. J. Dermatol..

[8] Peris K., Alaibac M., Argenziano G., Di Stefani A., Fargnoli M.C., Frascione P., Gualdi G., Longo C., Moscarella E., Naldi L. (2018). Cutaneous squamous cell carcinoma. Italian Guidelines by SIDeMaST adapted to and updating EADO/EDF/EORTC guidelines. G. Ital. Dermatol. Venereol..

[9] Fargnoli M.C., Piccioni A., Neri L., Tambone S., Pellegrini C., Peris K. (2017). Long-term efficacy and safety of daylight photodynamic therapy with methyl amninolevulinate for actinic keratosis of the face and scalp. Eur. J. Dermatol..

[10] Werner R.N., Sammain A., Erdmann R., Hartmann V., Stockfleth E., Nast A. (2013). The natural history of actinic keratosis: A systematic review. Br. J. Dermatol..

[11] Alam M., Ratner D. (2001). Cutaneous squamous-cell carcinoma. N. Engl. J. Med..

[12] Warner C.L., Cockerell C.J. (2011). The new seventh edition American Joint Committee on Cancer staging of cutaneous non-melanoma skin cancer: A critical review. Am. J. Clin. Dermatol..

[13] Pellegrini C., Orlandi A., Costanza G., Di Stefani A., Piccioni A., Di Cesare A., Chiricozzi A., Ferlosio A., Peris K., Fargnoli M.C. (2017). Expression of IL-23/Th17-related cytokines in basal cell carcinoma and in the response to medical treatments. PLoS ONE.

[14] Boukamp P. (2005). Non-melanoma skin cancer: What drives tumor development and progression?. Carcinogenesis.

[15] Forbes S.A., Beare D., Boutselakis H., Bamford S., Bindal N., Tate J., Cole C.G., Ward S., Dawson E., Ponting L. (2017). COSMIC: Somatic cancer genetics at high-resolution. Nucleic Acids Res..

[16] Pellegrini C., Maturo M.G., Martorelli C., Suppa M., Antonini A., Kostaki D., Verna L., Landi M.T., Peris K., Fargnoli M.C. (2017). Characterization of melanoma susceptibility genes in high-risk patients from Central Italy. Melanoma Res..

[17] Andrés-Lencina J.J., Rachakonda S., García-Casado Z., Srinivas N., Skorokhod A., Requena C., Soriano V., Kumar R., Nagore E. (2019). TERT promoter mutation subtypes and survival in stage I and II melanoma patients. Int. J. Cancer.

[18] Taylor R.S., Ramirez R.D., Ogoshi M., Chaffins M., Piatyszek M.A., Shay J.W. (1996). Detection of telomerase activity in malignant and nonmalignant skin conditions. J. Investig. Dermatol..

[19] Parris C.N., Jezzard S., Silver A., MacKie R., McGregor J.M., Newbold R.F. (1999). Telomerase activity in melanoma and non-melanoma skin cancer. Br. J. Cancer.

[20] Pellegrini C., Maturo M.G., Di Nardo L., Ciciarelli V., Gutierrez Garcia-Rodrigo C., Fargnoli M.C. (2017). Understanding the Molecular Genetics of Basal Cell Carcinoma. Int. J. Mol. Sci..

[21] Gunes C., Rudolph K.L. (2013). The role of telomeres in stem cells and cancer. Cell.

[22] Jafri M.A., Ansari S.A., Alqahtani M.H., Shay J.W. (2016). Roles of telomeres and telomerase in cancer, and advances in telomerase-targeted therapies. Genome Med..

[23] Nandakumar J., Cech T.R. (2013). Finding the end: Recruitment of telomerase to telomeres. Nat. Rev. Mol. Cell Biol..

[24] Rachakonda S., Kong H., Srinivas N., Garcia-Casado Z., Requena C., Fallah M., Heidenreich B., Planelles D., Traves V., Schadendorf D. (2018). Telomere length, telomerase reverse transcriptase promoter mutations, and melanoma risk. Genes Chromosomes Cancer.

[25] Szostak J.W., Blackburn E.H. (1982). Cloning yeast telomeres on linear plasmid vectors. Cell.

[26] Blackburn E.H. (1991). Structure and function of telomeres. Nature.

[27] Levy M.Z., Allsopp R.C., Futcher A.B., Greider C.W., Harley C.B. (1992). Telomere end-replication problem and cell aging. J. Mol. Biol..

[28] Harley C.B. (1991). Telomere loss: Mitotic clock or genetic time bomb?. Mutat. Res..

[29] Blackburn E.H. (2001). Switching and signaling at the telomere. Cell.

[30] Sharpless N.E., DePinho R.A. (2004). Telomeres, stem cells, senescence, and cancer. J. Clin. Investig..

[31] Xin H., Liu D., Songyang Z. (2008). The telosome/shelterin complex and its functions. Genome Biol..

[32] De Lange T. (2005). Shelterin: The protein complex that shapes and safeguards human telomeres. Genes Dev..

[33] Martinez P., Blasco M.A. (2011). Telomeric and extra-telomeric roles for telomerase and the telomere-binding proteins. Nat. Rev. Cancer.

[34] Kim N.W., Piatyszek M.A., Prowse K.R., Harley C.B., West M.D., Ho P.L., Coviello G.M., Wright W.E., Weinrich S.L., Shay J.W. (1994). Specific association of human telomerase activity with immortal cells and cancer. Science.

[35] Cesare A.J., Reddel R.R. (2010). Alternative lengthening of telomeres: Models, mechanisms and implications. Nat. Rev. Genet..

[36] Morin G.B. (1989). The human telomere terminal transferase enzyme is a ribonucleoprotein that synthesizes TTAGGG repeats. Cell.

[37] Greider C.W., Blackburn E.H. (1985). Identification of a specific telomere terminal transferase activity in Tetrahymena extracts. Cell.

[38] Chen Z., Smith K.J., Skelton H.G., Barrett T.L., Greenway H.T., Lo S.C. (2001). Telomerase activity in Kaposi’s sarcoma, squamous cell carcinoma, and basal cell carcinoma. Exp. Biol. Med..

[39] Pogacic V., Dragon F., Filipowicz W. (2000). Human H/ACA small nucleolar RNPs and telomerase share evolutionarily conserved proteins NHP2 and NOP10. Mol. Cell. Biol..

[40] Daniel M., Peek G.W., Tollefsbol T.O. (2012). Regulation of the human catalytic subunit of telomerase (hTERT). Gene.

[41] Akincilar S.C., Unal B., Tergaonkar V. (2016). Reactivation of telomerase in cancer. Cell. Mol. Life Sci..

[42] Hoffmeyer K., Raggioli A., Rudloff S., Anton R., Hierholzer A., Del Valle I., Hein K., Vogt R., Kemler R. (2012). Wnt/beta-catenin signaling regulates telomerase in stem cells and cancer cells. Science.

[43] Wong C.W., Hou P.S., Tseng S.F., Chien C.L., Wu K.J., Chen H.F., Ho H.N., Kyo S., Teng S.C. (2010). Kruppel-like transcription factor 4 contributes to maintenance of telomerase activity in stem cells. Stem Cells.

[44] Aviv A., Anderson J.J., Shay J.W. (2017). Mutations, Cancer and the Telomere Length Paradox. Trends Cancer.

[45] Ma H., Zhou Z., Wei S., Liu Z., Pooley K.A., Dunning A.M., Svenson U., Roos G., Hosgood H.D., Shen M. (2011). Shortened telomere length is associated with increased risk of cancer: A meta-analysis. PLoS ONE.

[46] Shay J.W., Wright W.E. (2011). Role of telomeres and telomerase in cancer. Semin. Cancer Biol..

[47] Wentzensen I.M., Mirabello L., Pfeiffer R.M., Savage S.A. (2011). The association of telomere length and cancer: A meta-analysis. Cancer Epidemiol. Biomark. Prev..

[48] Gramatges M.M., Telli M.L., Balise R., Ford J.M. (2010). Longer relative telomere length in blood from women with sporadic and familial breast cancer compared with healthy controls. Cancer Epidemiol. Biomark. Prev..

[49] Svenson U., Nordfjall K., Stegmayr B., Manjer J., Nilsson P., Tavelin B., Henriksson R., Lenner P., Roos G. (2008). Breast cancer survival is associated with telomere length in peripheral blood cells. Cancer Res..

[50] Zhang C., Doherty J.A., Burgess S., Hung R.J., Lindstrom S., Kraft P., Gong J., Amos C.I., Sellers T.A., Monteiro A.N. (2015). Genetic determinants of telomere length and risk of common cancers: A Mendelian randomization study. Hum. Mol. Genet..

[51] Wright W.E., Piatyszek M.A., Rainey W.E., Byrd W., Shay J.W. (1996). Telomerase activity in human germline and embryonic tissues and cells. Dev. Genet..

[52] Collins K., Mitchell J.R. (2002). Telomerase in the human organism. Oncogene.

[53] Allen N.D., Baird D.M. (2009). Telomere length maintenance in stem cell populations. Biochim. Biophys. Acta.

[54] Weise J.M., Gunes C. (2009). Differential regulation of human and mouse telomerase reverse transcriptase (TERT) promoter activity during testis development. Mol. Reprod. Dev..

[55] Kyo S., Takakura M., Fujiwara T., Inoue M. (2008). Understanding and exploiting hTERT promoter regulation for diagnosis and treatment of human cancers. Cancer Sci..

[56] Liu Z., Li Q., Li K., Chen L., Li W., Hou M., Liu T., Yang J., Lindvall C., Björkholm M. (2013). Telomerase reverse transcriptase promotes epithelial-mesenchymal transition and stem cell-like traits in cancer cells. Oncogene.

[57] Lagunas A.M., Wu J., Crowe D.L. (2017). Telomere DNA damage signaling regulates cancer stem cell evolution, epithelial mesenchymal transition, and metastasis. Oncotarget.

[58] El-Badawy A., Ghoneim N.I., Nasr M.A., Elkhenany H., Ahmed T.A., Ahmed S.M., El-Badri N. (2018). Telomerase reverse transcriptase coordinates with the epithelial-to-mesenchymal transition through a feedback loop to define properties of breast cancer stem cells. Biol. Open.

[59] Hanahan D., Weinberg R.A. (2011). Hallmarks of cancer: The next generation. Cell.

[60] Pellegrini C., Di Nardo L., Cipolloni G., Martorelli C., De Padova M., Antonini A., Maturo M.G., Del Regno L., Strafella S., Micantonio T. (2018). Heterogeneity of BRAF, NRAS, and TERT Promoter Mutational Status in Multiple Melanomas and Association with MC1R Genotype: Findings from Molecular and Immunohistochemical Analysis. J. Mol. Diagn..

[61] Nagore E., Heidenreich B., Requena C., García-Casado Z., Martorell-Calatayud A., Pont-Sanjuan V., Jimenez-Sanchez A.I., Kumar R. (2016). TERT promoter mutations associate with fast-growing melanoma. Pigment Cell Melanoma Res..

[62] Pópulo H., Boaventura P., Vinagre J., Batista R., Mendes A., Caldas R., Pardal J., Azevedo F., Honavar M., Guimarães I. (2014). TERT promoter mutations in skin cancer: The effects of sun exposure and X-irradiation. J. Investig. Dermatol..

[63] Vinagre J., Pinto V., Celestino R., Reis M., Populo H., Boaventura P., Melo M., Catarino T., Lima J., Lopes J.M. (2014). Telomerase promoter mutations in cancer: An emerging molecular biomarker?. Virchows Arch..

[64] Kumar R., Khan R., Gupta N., Seth T., Sharma A., Kalaivani M., Sharma A. (2018). Identifying the biomarker potential of telomerase activity and shelterin complex molecule, telomeric repeat binding factor 2 (TERF2), in multiple myeloma. Leuk. Lymphoma.

[65] Haycock P.C., Burgess S., Nounu A., Zheng J., Okoli G.N., Bowden J., Wade K.H., Timpson N.J., Evans D.M., The Telomeres Mendelian Randomization Collaboration (2017). Association Between Telomere Length and Risk of Cancer and Non-Neoplastic Diseases: A Mendelian Randomization Study. JAMA Oncol..

[66] Ribero S., Mangino M., Bataille V. (2016). Skin phenotypes can offer some insight about the association between telomere length and cancer susceptibility. Med. Hypotheses.

[67] Horn T. (2008). Comments on quantitative real-time PCR for measurement of telomere length. Cancer Investig..

[68] Bataille V., Kato B.S., Falchi M., Gardner J., Kimura M., Lens M., Perks U., Valdes A.M., Bennett D.C., Aviv A. (2007). Nevus size and number are associated with telomere length and represent potential markers of a decreased senescence in vivo. Cancer Epidemiol. Biomark. Prev..

[69] Campbell P.J. (2012). Telomeres and cancer: From crisis to stability to crisis to stability. Cell.

[70] Han J., Qureshi A.A., Prescott J., Guo Q., Ye L., Hunter D.J., De Vivo I. (2009). A prospective study of telomere length and the risk of skin cancer. J. Investig. Dermatol..

[71] Wainwright L.J., Middleton P.G., Rees J.L. (1995). Changes in mean telomere length in basal cell carcinomas of the skin. Genes Chromosomes Cancer.

[72] Griewank K.G., Murali R., Schilling B., Schimming T., Moller I., Moll I., Schwamborn M., Sucker A., Zimmer L., Schadendorf D. (2013). TERT promoter mutations are frequent in cutaneous basal cell carcinoma and squamous cell carcinoma. PLoS ONE.

[73] Toll A., Salgado R., Yebenes M., Martin-Ezquerra G., Gilaberte M., Baro T., Sole F., Alameda F., Espinet B., Pujol R.M. (2009). MYC gene numerical aberrations in actinic keratosis and cutaneous squamous cell carcinoma. Br. J. Dermatol..

[74] Desmaze C., Soria J.C., Freulet-Marriere M.A., Mathieu N., Sabatier L. (2003). Telomere-driven genomic instability in cancer cells. Cancer Lett..

[75] Perrem K., Lynch A., Conneely M., Wahlberg H., Murphy G., Leader M., Kay E. (2007). The higher incidence of squamous cell carcinoma in renal transplant recipients is associated with increased telomere lengths. Hum. Pathol..

[76] Leufke C., Leykauf J., Krunic D., Jauch A., Holtgreve-Grez H., Bohm-Steuer B., Brocker E.B., Mauch C., Utikal J., Hartschuh W. (2014). The telomere profile distinguishes two classes of genetically distinct cutaneous squamous cell carcinomas. Oncogene.

[77] Yamada-Hishida H., Nobeyama Y., Nakagawa H. (2018). Correlation of telomere length to malignancy potential in non-melanoma skin cancers. Oncol. Lett..

[78] Caini S., Raimondi S., Johansson H., De Giorgi V., Zanna I., Palli D., Gandini S. (2015). Telomere length and the risk of cutaneous melanoma and non-melanoma skin cancer: A review of the literature and meta-analysis. J. Dermatol. Sci..

[79] Liang G., Qureshi A.A., Guo Q., De Vivo I., Han J. (2011). No association between telomere length in peripheral blood leukocytes and the risk of nonmelanoma skin cancer. Cancer Epidemiol. Biomark. Prev..

[80] Anic G.M., Sondak V.K., Messina J.L., Fenske N.A., Zager J.S., Cherpelis B.S., Lee J.H., Fulp W.J., Epling-Burnette P.K., Park J.Y. (2013). Telomere length and risk of melanoma, squamous cell carcinoma, and basal cell carcinoma. Cancer Epidemiol..

[81] Ueda M., Ouhtit A., Bito T., Nakazawa K., Lubbe J., Ichihashi M., Yamasaki H., Nakazawa H. (1997). Evidence for UV-associated activation of telomerase in human skin. Cancer Res..

[82] Boldrini L., Loggini B., Gisfredi S., Zucconi Y., Di Quirico D., Biondi R., Cervadoro G., Barachini P., Basolo F., Pingitore R. (2003). Evaluation of telomerase in non-melanoma skin cancer. Int. J. Mol. Med..

[83] Burnworth B., Arendt S., Muffler S., Steinkraus V., Brocker E.B., Birek C., Hartschuh W., Jauch A., Boukamp P. (2007). The multi-step process of human skin carcinogenesis: A role for p53, cyclin D1, hTERT, p16, and TSP-1. Eur. J. Cell Biol..

[84] Scott G.A., Laughlin T.S., Rothberg P.G. (2014). Mutations of the TERT promoter are common in basal cell carcinoma and squamous cell carcinoma. Mod. Pathol..

[85] Campos M.A., Macedo S., Fernandes M., Pestana A., Pardal J., Batista R., Vinagre J., Sanches A., Baptista A., Lopes J.M. (2019). TERT promoter mutations are associated with poor prognosis in cutaneous squamous cell carcinoma. J. Am. Acad. Dermatol..

[86] Cheng K.A., Kurtis B., Babayeva S., Zhuge J., Tantchou I., Cai D., Lafaro R.J., Fallon J.T., Zhong M. (2015). Heterogeneity of TERT promoter mutations status in squamous cell carcinomas of different anatomical sites. Ann. Diagn. Pathol..

[87] Nan H., Qureshi A.A., Prescott J., De Vivo I., Han J. (2011). Genetic variants in telomere-maintaining genes and skin cancer risk. Hum. Genet..

